# Computational analysis of efficient organic solar cell-based retinal prosthesis using plasmonic gold nanoparticles

**DOI:** 10.3389/fncel.2023.1205048

**Published:** 2023-07-27

**Authors:** Ali Rahmani, Kyungsik Eom

**Affiliations:** ^1^Department of Electronics Engineering, College of Engineering, Pusan National University, Busan, Republic of Korea; ^2^Department of Electronics, College of Electrical and Computer Engineering, Yadegar-e-Imam Khomeini (RAH) Shahre-e-Rey Branch, Islamic Azad University, Tehran, Iran

**Keywords:** organic solar cell, spherical gold nanoparticles, localized surface plasmon resonance, photovoltaic retinal prosthesis, neural interface

## Abstract

**Introduction:**

Photovoltaic restoration of vision, especially in conjunction with the use of silicon photodiodes, has gained attention for use in patients affected by blindness due to retinal layer disease. Although the use of silicon photodiodes offers miniaturization of the implant unit and increase in the stimulation channel, the implant unit may suffer from the fracture of these brittle photodiodes when mechanical pressure exerted.

**Methods:**

We present an organic solar cell (OSC)-based retinal prosthesis in which spherical gold nanoparticles (AuNPs) are embedded into the active layer to increase the efficiency of the bioelectric interface.

**Results:**

We demonstrate computationally that a modeled OSC incorporating spherical AuNPs has three times higher efficiency than that of a bare OSC presented before for retinal prostheses. Our AuNP based OSC was able to activate the neuron at the minimum light intensity of 0.26 mW/mm^2^, which is lower than that of the bare OSC.

**Discussion:**

The use of AuNPs in OSC allows device miniaturization or lowering of the light exposure required for neural activation using a photovoltaic retinal prosthesis, which can generally be applied in a broad range of neural prostheses.

## 1. Introduction

Age-related macular degeneration and retinitis pigmentosa are the most prevalent incurable eye diseases leading to photoreceptor degeneration and irreversible blindness (Wong et al., 2014). Age-related macular degeneration is the primary cause of irreversible vision loss in the elderly affecting 1.5% of Americans over 40 to >15% of those over 80, while retinitis pigmentosa affects younger people and leads to more vision loss (Palanker et al., 2022). Retinal prostheses provide a way to restore vision in blind patients suffering from retinal diseases by artificially stimulating the surviving retinal neural networks, such as bipolar cells and retinal ganglion cells (RGCs) (Lorach et al., 2015).

Microelectrode arrays inserted into the epiretinal and subretinal spaces have been extensively studied for the electrical activation of retinal neurons to restore light sensation (Mandel et al., 2013). Argus II (Second Sight Medical Products, Inc., CA, United States) was the first epiretinal implant approved by the Food and Drug Administration that targeted RGCs with a visual acuity below 20/1,260 (Arevalo et al., 2021). Implantation of a large implant unit and the unavoidable connection from the receiver coil to the electrode array require sophisticated surgical procedures and increase the likelihood of adverse effects (DeMarco et al., 2007). In addition, perceived vision is entirely dependent on the camera’s direction rather than on eye movement. The Alpha IMS (Retina Implant AG, Reutlingen, Germany) is one of the most successful subretinal implants that stimulate the inner nuclear layer of the retina (Stingl et al., 2015). In contrast to Argus II, data delivery via an inductive link is not required as silicon photodiodes are implanted to capture the external scene, thereby increasing the number of electrodes (Klauke et al., 2011). A subretinally implanted silicon photodiode was illuminated by a high-intensity stimulation light pattern projected outside the eye, which converted light energy to electrical current to stimulate the inner nuclear layer. The use of a photodiode in combination with an external light projection minimizes the size of the implanted unit. Moreover, the use of pixels with widths equal to 100 μm in the wireless photovoltaic retinal implant (PRIMA; Pixium Vision, Paris, France) allows improved visual acuity ranging from 20/550 to 20/460 (Palanker et al., 2022). Despite these remarkable accomplishments, current wireless photovoltaic retinal prostheses face significant challenges in terms of potential safety. Because these prostheses are fabricated on a silicon substrate, they may fracture when exposed to the mechanical pressure. Moreover, being implanted in a curved eyeball, these prostheses are continuously subjected to mechanical bending forces that damage the retinal layer. Therefore, thin, and flexible photovoltaic devices are desirable which have less concern to the exerted mechanical stress specially during the surgery and implanting the interface (Antognazza et al., 2016).

As effective photovoltaic devices in neuronal prostheses, organic materials have been proven to be successful in converting light signals to electrical voltage to stimulate neurons (Shim et al., 2020). In recent decades, many research groups have focused on improving the quality and efficiency of organic interfaces, thus making them more widely applicable and cost-effective (Ghezzi et al., 2011; Kelly et al., 2011; Hadjinicolaou et al., 2012). Organic materials maintain their activity when in contact with electrolyte solutions, and neurons can grow effectively on these materials (Antognazza et al., 2009). In addition, organic layers in the subretinal configuration can restore the light sensitivity of retinas extracted from retina-degenerated rats (Feyen et al., 2016). The use of organic materials offers many advantages over silicon devices, including low cost, lightweight, high flexibility, and bendability of neural interface to form in retinal curvature (Ghezzi et al., 2011). However, several issues remain unresolved. One crucial issue is that the low-photoconversion efficiency of these organic devices prevents them from shrinking, thus making it difficult for them to achieve large channel densities (Pillai and Green, 2010). Another issue is the requirement for high-output light intensity to stimulate retinal neurons, which may damage the retinal tissue (Mathieson et al., 2012). Many researchers have developed some conjugated polymers, such as poly(3-hexylthiophene-2,5-diyl), P3HT; [poly(3,4-ethylenedioxythiophene)-polystyrene sulfonate], PEDOT:PSS, and other organic semiconductors ([6,6]-phenyl-C61-butyric acid methyl ester, PC_61_BM) as photovoltaic subretinal prostheses to enhance visual acuity (Antognazza et al., 2016; Maya-Vetencourt et al., 2017). Other groups have presented epiretinal prostheses (POLYRETINA) using the P_3_HT:PC_60_BM bulk heterojunction (BHJ), which was able to activate RGCs (Ferlauto et al., 2018); however, given the poor photoconversion of organic solar cells (OSCs) made of pure BHJ, either the cell size or projected light intensity should be increased, limiting the stimulation resolution or safety issues, respectively (Rathbun et al., 2014).

The main factors limiting the power conversion efficiency (PCE) of the OSC are low-light absorption and low-photogenerated exciton extraction efficiency (Jung et al., 2015). To enhance the PCE of OSCs, numerous light-trapping materials, such as quantum dots (Wu et al., 2015) and nanowires (Christesen et al., 2012), and plasmonic nanoparticles (NPs) have been employed. The use of plasmonic metal nanoparticles is a practical strategy for boosting light absorption in an organic active layer without sacrificing the electrical properties or degrading the device structure (Tan et al., 2012). In some studies, plasmonic metal NPs were located both inside and outside the organic active layers (Wang et al., 2018).

In this study, we investigated theoretically an efficient photovoltaic retinal prosthesis based on organic materials, using spherical gold NPs (AuNPs) in an active P_3_HT:PCBM layer. In contrast to the presented bare active layer solar cells which require high light intensity or have a low PCE, this type of solar cell exploits the lower light intensity or higher PCE. For this purpose, regarding the retinal prostheses considerations, several factors, such as the size and filling fraction (f_s_) of the NPs and their effects on parameters, such as the current density, absorption, and scattering terms in the active layer, were investigated. The incident light was tuned to a wavelength of 610 nm, which caused high-light absorption due to the localized surface plasmon resonance (LSPR) wavelength of the AuNPs. We modeled the active layer cell of the BHJ as a mixed donor-acceptor layer intertwined in a comb shape. The cell interface was assumed to be curved to be closer to the actual structure. We considered both the absorption and scattering cross-sections of the NPs to estimate the entire effective region of exciton generation in the active layer in the COMSOL simulator. We computationally modeled the neuron and incorporated it into a NPs-embedded solar cell to determine the optical intensity threshold required to activate the neuron.

## 2. Materials and methods

### 2.1. Solar cell modeling

Organic solar cells usually include different organic or inorganic layers. The active absorbing layer is an organic material in which free charges are generated (Sompech et al., 2016). This device comprises two electrodes on both sides of the active layer (anode and cathode), one of which is transparent (Fleetham et al., 2015). In inorganic materials, thermal energy (K_B_T ≈ 26 meV) is sufficient to dissociate excitons into free charges. However, in organic compounds, the binding energy is high (approximately 0.5 eV). Therefore, their separation requires the blend of the two materials with the necessary energy and the formation of a BHJ.

The performance of OSCs is based on the mechanism of the electron donor and acceptor, which utilizes the advantages of the BHJ structure to broaden the interface between the donor and acceptor in the active layer. An increased interface enhances the probability of charge-pair dissociation and prevents recombination phenomena that result in a high PCE (Shabani et al., 2022). Therefore, in contrast to other active-layer structures, such as single-layer, bilayer, and laminated layers, the BHJ is the optimal choice for a bare active layer (Zidan et al., 2021). Because of their ability to stimulate the neurons, a mixture of P_3_HT and PCBM has proven appealing compared with other organic materials (Zidan et al., 2021; Shabani et al., 2022). The charge carrier mobility and exciton diffusion length in organic semiconductors are much smaller than in inorganic crystalline materials (Longeaud et al., 2016). Thus, a trade-off between a high thickness for light absorption and a low thickness for exciton dissociation should be considered to enhance the photoconversion efficiency. The balanced thickness of the organic photoactive layers was in the range of 100 nm for the active layer (Ma et al., 2005).

**FIGURE 1:**
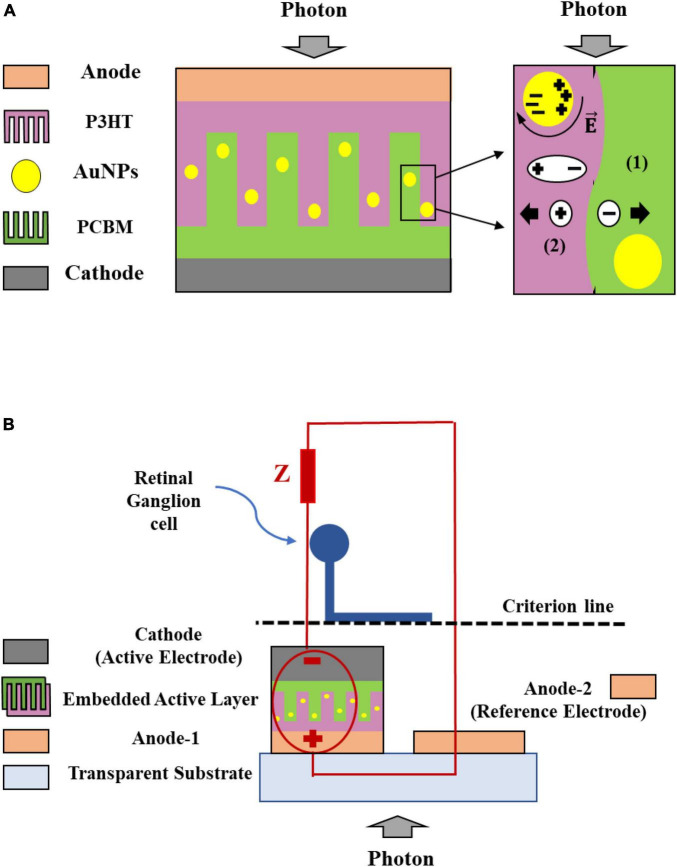
Gold nanoparticles (AuNPs)-embedded solar cell structure and the process of the exciton (1), and free carrier generation (2), considering the comb shape of the active layer with a curvature interface between the donor and acceptor **(A)**. The solar cell was used as a voltage source to stimulate the unmyelinated neurons (circuit model) **(B)**.

The BHJ active layer was modeled in two dimensions (Figures 1A, B). The blended BHJ architecture can be modeled as a periodic comb shape in which the periodicity allows us to simulate only a segment of the structure (Raba et al., 2013). Because the contact surface of the donor and acceptor increases in the BHJ blend, and their boundaries intertwine with each other, the comb shape is a suitable approximation for modeling the active layer. Herein, we assumed that the interface between the two domains was non-flat and considered the curvature form to be closer to the actual structure. L, W_d_, and W_a_ denote the active-layer thickness, and mean width of the donor and acceptor domains, respectively. The donor to acceptor ratio (D/A) is determined by considering the densities of P_3_HT and PCBM (Prosa et al., 1992). For the blend of P_3_HT:PCBM with a 1:0.8 weight ratio, the volume ratio was approximately 1.4. Considering the mechanism governing the generation and diffusion of excitons toward the interface and charge transfer states, and using the mathematical description of the system, the main parameters of the modeled structure can be determined (Raba et al., 2013). Accordingly, the mean widths of the donor and acceptor were 14 and 10 nm, respectively, and the thickness of the active layer, L, was assumed to be 100 nm.

The photovoltaic structure was simulated using the finite element analysis software COMSOL Multiphysics (COMSOL Inc., Burlington, MA, USA). In the simulation, we used two-dimensional domain, semiconductor module, and fine meshing. The simulated structure is the active layer cell (Figure 1A) that includes two layers of P_3_HT and PCBM as the donor and acceptor layers, respectively, with a curved interface. In this simulation, all materials were selected from the COMSOL library, and the complex refractive indices of the materials were inserted as a function of the incident light wavelength. Figure 1B shows the solar cell (circuit model) which stimulates the neurons as a voltage source.

### 2.2. Modeling of plasmonic AuNPs

Metal NPs have been employed in buffer layers, active layers, and at the interface of OSCs to enhance light absorption and increase current density (Su et al., 2012). The incorporation of NPs improves light harvesting through LSPR via two mechanisms: near-field plasmonic interactions and forward light scattering, thus resulting in an increase in the optical path length. When NPs are embedded in the active layer, strong local field enhancements occur in the vicinity of the NPs owing to resonant plasmon excitation. Excitons are generated in these strong fields, thus causing increased light absorption and high PCE. Among the various metals, gold was chosen because it is inert in biological tissues.

For metal NPs smaller than the wavelength of the incident light, the absorption (C_abs_) and scattering (C_sc_) cross-sectional areas from the quasi-static approximation are expressed by the following equations (Bohren, 1983):


(1) $$C_{a\,b\,s}=\frac{2\,\pi }{\lambda }\,I\,m\,\left[\alpha_{s\,p}\right]$$

(2) $$C_{s\,c\,a\,t}=\frac{1}{6\,\pi }\,{\left(\frac{2\,\pi }{\lambda }\right)}^{4}\,{\left|\alpha_{s\,p}\right|}^{2}$$

(3) $$\alpha_{s\,p}=3\,V\,\frac{\varepsilon_{m}-\varepsilon_{s}}{\varepsilon_{m}+2\,\varepsilon_{s}}$$

where α_*sp*_ is the polarizability of the NPs, *V* is the NP volume, *r* is the radius of NP, λ is the incident light wavelength, and ε_*m*_ and ε_*s*_ are wavelength dependent dielectric functions of the metal NPs and surrounding medium, respectively. The normalized absorption (Q_abs_) and scattering cross-section (Q_scat_) are calculated by the ratio of their terms and the cross-sectional area of NPs, which is πr^2^, i.e., Q_abs_ is equal to C_abs_/πr^2^ and Q_scat_ is equal to C_scat_/πr^2^. The scattering efficiency is given by Bohren (1983),


(4) $$Q_{s\,c}=\frac{C_{s\,c\,a\,t}}{C_{s\,c\,a\,t}+C_{a\,b\,s}}$$

Scattering efficiency indicates the proportion of scattering relative to the absorption cross-sectional area. In resonance conditions, wherein the frequency of the incident light is close to the frequency of the surface plasmon resonance, the denominator (Equation 3) equals zero. This resulted in an increased polarizability of the NPs, enabling the incident light to interact with a larger area than the cross-section of the NP. Consequently, a thorough investigation of the impact of NPs on the absorption and scattering cross-sections, as well as the scattering efficiency, was conducted to improve the interaction between the incident light and the NPs.

We first set the geometry of the NPs, wavelength of the incident light, and material properties of the NPs and surrounding medium, as they determine the scattering and absorption cross-sections. Gold and P_3_HT:PCBM were selected as materials for the NPs and their surroundings, respectively. Concerning the geometry of the AuNPs, a spherical shape was chosen because of its strong LSPR phenomenon when illuminated with visible light. The radius of the spherical AuNPs was constrained to 10 nm because the scattering effect was overwhelmed by their absorption for sizes beyond this limit. Moreover, exciton quenching and carrier recombination reduce the PCE of larger AuNPs (Li et al., 2020). Three different AuNP sizes (5, 7.5, and 10 nm) were designed and simulated.

### 2.3. Current generation in the solar-embedded AuNPs

Metal nanoparticles with nanoscale dimensions have significant effects on free-carrier generation owing to their unusual optoelectronic properties in OSCs (Su et al., 2012). When the AuNPs were under resonant light wavelength, strong interactions were observed between the free carriers in the AuNPs and the incident electromagnetic field. The collective oscillations of the free electrons were plasmons, followed by localized dipole production in the proximity of the AuNP surface. The fields created by these localized dipoles generate excitons in the active organic layer. Free electrons arising from these excitons at the interface cause higher current generation compared with the bare active layer (Wang et al., 2011).

The current density of the device was calculated by introducing the generation rate (G) of free carriers into the COMSOL simulator. The generation rate and short-circuit current density (J_sc_) equations for the bare active layer are defined by Equations 5, 6, respectively (Wu et al., 2011; Piralaee et al., 2020). The short-circuit current density (J_sc_) of a solar cell is the current density in short-circuit conditions.


(5) $$G\,\left(\lambda \right)=\alpha \,\left(\lambda \right)\,\lambda \,I_{0}\,P/h\,c$$

(6) $$J_{s\,c}\,\left(\lambda \right)=G\,\left(\lambda \right)\,q\,L$$

where α is the absorption coefficient and equals $\frac{4\,\pi }{\lambda }\,k\,\left(\lambda \right)$, *k* is the imaginary part of the medium refraction index, λ is the incident light wavelength, *I*_0_ is light intensity, *h* is Planck’s constant, *c* is the speed of the light, *P* is the probability of excitons dissociation at the interface, *q* is the electron charge, and *L* is the cell’s height.

For the active layers with AuNPs, the total J_sc_ was modified to consider not only the free carrier generation in the bare active layer but also the effects of absorption and scattering of light at the AuNPs in the active layer. Using Equations 5, 6 and considering *P* in Equation 5 as the probability of exciton dissociation in the medium from the presented experimental works, this parameter is 79.2% for the bare active layer and 84.4% for AuNPs-embedded active layer (Wu et al., 2011); the short circuit current density equation is described as,


(7) $$J_{s\,c}\left(\lambda \right)=\left(I_{0}\lambda qL/hc\right)[\left(79.2\%\alpha_{1}\left(\lambda \right)\left(1-f_{s}-v_{1}f_{s}-v_{2}f_{s}\right)\right)$$

$$+\left(84.4\%\alpha_{2}\left(\lambda \right)\left(1-Q_{s\,c}\right)\left(v_{1}f_{s}\right)\right)+\left(84.4\%\alpha_{2}\left(\lambda \right)Q_{s\,c}\left(v_{2}f_{s}\right)\right)]$$

where α_1_ and α_2_ are absorption coefficients as a function of the incident light wavelength for the bare P_3_HT:PCBM and AuNPs, respectively. *Q*_*sc*_ is the scattering efficiency, *f_s_*, *v_1_f_s_*, and *v_2_f_s_* show the fraction of NPs, absorption term, and the scattering term in the active layer volume, respectively. v_1_ is the ratio of v_abs_ to v_NP_, where v_abs_ is the volume of the absorption cross-section (near-field absorption region around the NP), and v_NP_ is the volume of the NP. In addition, v_2_ is the ratio of v_scat_ to v_NP_, where v_scat_ is the volume of the scattering cross-section (the scattering region around the NP). The first term of the generation rate of free carriers is the fraction of the generated free carriers in the active layer, whereas the second term represents the generated free carriers owing to the light absorption of AuNPs with a probability of (1 − Q_sc_). The scattering efficiency (Q_sc_) determines the ratio of the scattering and absorption cross-sections in free carrier generation. The third term generates free carriers owing to the scattering cross-section of AuNPs with the probability of Q_sc_ in the short-circuit current density. The modeling parameters of the solar cells and their values are listed in Table 1.

**TABLE 1:** Modeling parameters and their values for the solar cell in COMSOL.

Parameter	Definition	Value	Unit
r	AuNPs radius	5, 7.5, and 10	nm
f_s_	Filling fraction	10, 15, and 20%	–
I	Incident light intensity	0.26 and 0.38	mW/mm^2^
A	Cell area	100 × 100	μm^2^
D	Active layer thickness	100	nm
W_d_	Donor thickness	14	nm
W_a_	Acceptor thickness	10	nm

### 2.4. Plasmonic solar cell-based neural stimulation system

We intended to design an OSC and identify a way to increase its PCE by varying the size and filling fraction of AuNPs. We validated whether our modeled organic solar radiation could stimulate neurons and determined the required optical intensity for neural activation. To achieve this, we modeled a neuron with stimulating electrodes that could generate an electrical stimulation pulse generated by a solar cell using the neuronal dynamics simulator SIM4LIFE LIGHT [Zurich Medtech AG (ZMT), Switzerland].

The architecture of the neural stimulation system using a plasmonic solar cell in which the AuNPs were embedded in the active layer is shown in Figure 1. Following a previously demonstrated retinal prosthesis with an electrode size of 100 μm×100μm (Ferlauto et al., 2018), we designed a pixel with the same size (A). Each pixel included a cathode gate, a blend of P_3_HT:PCBM semiconductor as the active layer with a thickness (D) of 100 nm, and an electron-blocking layer as the anode at the bottom part.

To model the unmyelinated neurons, a spatially extended stimulation model was employed. This neuroelectric model was used to simulate an effective electrical nerve stimulation. To inject current into the neuron using a voltage source with electrical characteristics identical to those of the designed solar cell, we modeled the electrodes and neuron. In this structure, the active and reference electrode areas (A) with a thickness (t) of 150 nm from titanium (Ti), a metal which primarily delivers charge through the capacitive process, is typical option used in many experimental studies (Ferlauto et al., 2018). The RGC was positioned precisely atop the central region of the active electrode, parallel to the electrode plate with a distance (l) of 5 μm from the surface of the electrode (Figure 1B). The axon’s diameter (d) was deliberately chosen as 1 μm, falling within the diameter range observed in human retinal axons (FitzGibbon and Taylor, 2012; FitzGibbon and Nestorovski, 2013). Monophasic cathodic stimulation was employed, and the voltage magnitude was swept to determine the threshold voltage. The modeling parameters of the neurons and solar cells and their values are listed in Table 2.

**TABLE 2:** Modeling parameters and their values for neurons in SIM4LIFE LIGHT.

Parameter	Definition	Value	Unit
A	Electrode area	100 × 100	μm^2^
t	Electrode thickness	150	nm
l	Neuron distance from electrode surface	5	μm
d	Axon diameter	1	μm

**FIGURE 2:**
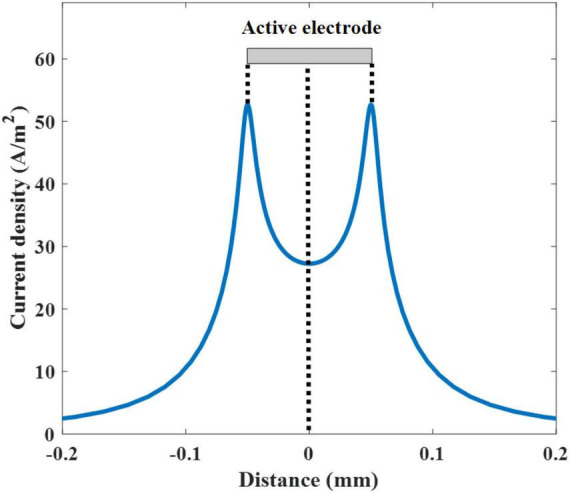
Threshold current density used to stimulate the neuron as a function of the distance along the electrode. The active electrode is presented as a gray-colored rectangle. The center of the active electrode is set to 0 mm.

As the electrode in the solar cell interfaces with the neuron, the current injected into the neuron at a given applied voltage is determined by the impedance of the electrode to the neuron (Figure 1B). In this case, the electrode is assumed as a perfect contact and thereby the steady state current is injected during the stimulation. The spatial distributions of the electric potential and current density were calculated at the frequency of 10 Hz. The calculated electric currents were then applied to the neuron with a cathodic duration of 50 ms, which is identical to the 10 Hz sinusoidal stimulus. The threshold current density is determined by increasing the input pulse until the neuron triggers an action potential. The threshold current density is plotted against the distance along the active electrode whose center was fixed at 0 mm. We measured the current density along the transverse direction parallel to the surface of the electrode on the axonal trajectory (criterion line), 5 μm above the electrode surface (Figure 2). The average current density from edge to edge of the active electrode (−0.05 to +0.05 mm) was 33.63 A/m^2^. Two spikes (at −0.05 and +0.05 mm) were observed at the edge of the active electrode. Regarding the given threshold voltage and current density which are 0.065 V and 33.63 A/m^2^, respectively, the total impedance across the electrode-to neuron is calculated as V/I. The values of the total impedance in 10 and 100 Hz are 1.9 and 0.57 kΩ mm^2^, respectively. This trend shows that increasing the simulation frequency reduces the total impedance. In this case, based on the SIM4LIFE material library, the tissue electric conductivity and relative permittivity values in 10 Hz are 0.027 S/m and 4.06 × 10^7^, and these values in 100 Hz are 0.089 S/m and 3.90 × 10^6^, respectively. The calculated total impedance was used to determine the optical threshold intensity for photovoltaic neural stimulation.

## 3. Results

### 3.1. Localized surface plasmon resonance of AuNPs

To enhance the light absorption and PCE of the conventional bare active layer (e.g., P_3_HT:PCBM)-based solar cells, plasmonic spherical AuNPs were embedded in the active layer. These AuNPs serve as light-trapping centers that can enhance the photon absorption through the near-field absorption and far-field scattering via increasing the optical path length. The photon absorption and scattering spectra of solar cells with AuNPs in the active layer were investigated in terms of the wavelength of the incident light. The fractions of absorption and scattering in the light-trapping process are important for calculating the current generation parameters (i.e., v_1_ and v_2_ in Equation 7) and were assessed while the size of the AuNPs was varied. Other solar cell properties, such as current generation, current and voltage (I–V) characteristics, and power–voltage relations for different sizes and f_s_ of AuNPs, were investigated to modify and determine the optical characteristics of the solar cell.

**FIGURE 3:**
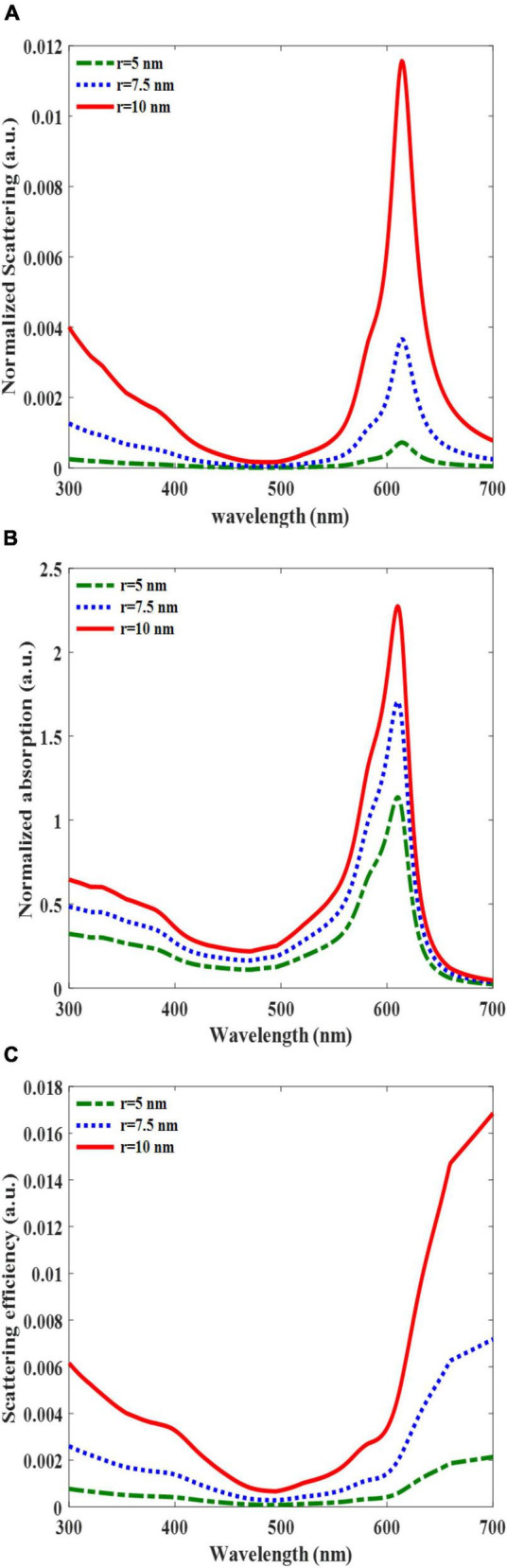
Normalized scattering **(A)**, normalized absorption cross-section **(B)**, and scattering efficiency **(C)**, for embedded AuNPs in the active layer versus the incident light wavelength for different AuNP radii were all equal to 5, 7.5, and 10 nm.

The normalized scattering and absorption cross-sections over the incident light wavelength revealed that the scattering and absorption reached a maximum value at a resonant frequency of 610 nm and minimum at approximately 500 nm (Figures 3A, B). The peak values of the scattering and absorption terms increased as the radius of the AuNPs increased. This is attributed to the dependence of these terms on the polarizability of the AuNPs, which is directly related to the AuNP volume (Equation 3). Interestingly, the normalized absorption cross-section overwhelms the scattering term for the entire simulated wavelength. The peak values of the normalized absorption cross-sections with AuNPs radii of 10, 7.5, and 5 nm were 2.3, 1.7, and 1.2, respectively, while the peaks of the normalized scattering term of 10, 7.5, and 5 nm were 0.0116, 0.0037, and 0.0008, respectively. These outcomes demonstrate that the contribution of light trapping by AuNPs is limited owing to their absorption. The scattering term was dominant only for AuNPs with radii larger than 25 nm (Li et al., 2020). In additional analyses that showed the variation of the scattering efficiency as a function of wavelength (Figure 3C), we also confirmed that the scattering efficiency, the portion of scattering over the total absorption and scattering term, was limited up to 0.016, which is the maximum value of the entire simulated case. The scattering efficiency decreased as the AuNP size decreased. Although total photon trapping can be boosted by the scattering of large AuNPs, this enhancement cannot compensate for the PCE reduction. This is owing to the fact that as the size of AuNPs increases in the active layer, exciton quenching and charge recombination deteriorate the PCE (You et al., 2014).

### 3.2. Effects of AuNPs on solar cell performance

Having found that AuNPs significantly promote light trapping by the LSPR phenomena, especially by means of absorption by the LSPRs effect, it is essential to validate whether light trapping via AuNPs can enhance current generation at the active layer. In this study, we evaluated the effect of the size of AuNPs and their relative concentration inside the active layer on the current generation of solar cells.

**FIGURE 4:**
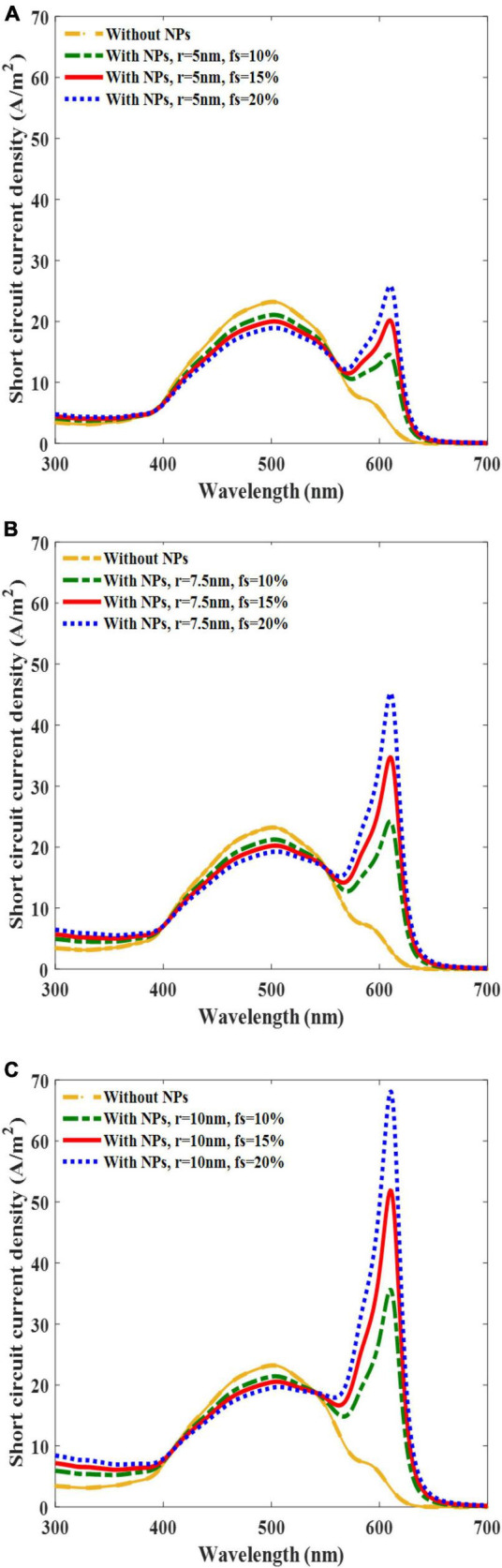
Plots of solar cell short-circuit current density versus incident light wavelength at the incident light intensity of 0.26 mW/mm^2^ and at the different filling fraction (f_s_) of gold in active layer volumes of 10, 15, and 20% and different radii equal to 5 nm **(A)**, 7.5 nm **(B)**, and 10 nm **(C)**.

To evaluate the effects of AuNPs on the current generation, the short-circuit current densities of the solar cells with and without AuNPs were investigated. When a solar cell with a bare active layer was irradiated with a light intensity of 0.26 mW/mm^2^, the maximum short-circuit current density was found to be approximately 500 nm (Figure 4). Interestingly, another sharp current peak was observed near a wavelength of 610 nm for the solar cell with AuNPs, which originated from the LSPRs of the AuNPs, especially owing to light absorption. These current peaks at 610 nm increased as the size of the AuNPs or f_s_ increased, thus indicating that as the radius of the AuNPs increased, the short-circuit current density originating from absorption and scattering increased, but by different amounts (Equation 7). Unlike the case at 610 nm, an increase in f_s_ led to a decrease in the short-circuit current density at 500 nm. We conjecture that this change arises from a reduction in the volume fraction of the active layers as f_s_ increases.

An enhancement in the short-circuit current density at a wavelength of 610 nm relative to 500 nm was observed in both the 7.5 and 10 nm AuNP-incorporated solar cells for all f_s_. Notably, we observed an increase in the short-circuit current density at 610 nm compared with that at 500 nm in both the 7.5 and 10 nm AuNP-integrated solar cells for all f_s_ values (Figures 4B, C).

However, for 5 nm AuNPs, only the f_s_ value of 20% yielded photocurrent at 610 nm which were larger by 1.1. times compared with those at 500 nm, while f_s_ values smaller than 20% yielded a lower photocurrent at 610 nm compared with that of 500 nm (Figure 4A). Taken together, these results suggest that to achieve the maximum photocurrent, it is advantageous to use AuNPs with sizes ≥7.5 nm and adjust the incident light wavelength to 610 nm. This is because of the higher absorption cross-section of larger AuNPs, and hence owing to the higher free carrier generation at the resonant wavelength.

**FIGURE 5:**
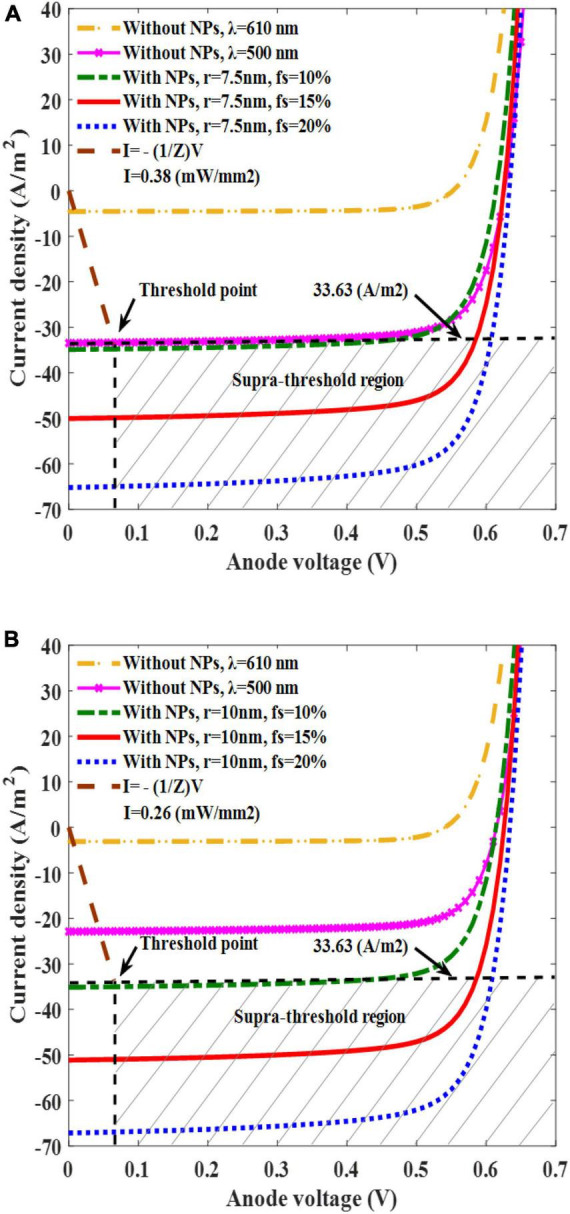
Plots of solar cell current density versus anode voltage for the filling fractions (f_s_) of 10, 15, and 20% for a AuNP radius of 7.5 nm and light intensity of 0.38 mW/mm^2^ **(A)**, and for an AuNP radius of 10 nm and a light intensity of 0.26 mW/mm^2^ **(B)**.

After confirming the enhanced current generation with the aid of AuNPs, especially for the sizes of 7.5, and 10 nm, we then simulated the I–V response of solar cells with AuNPs for various f_s_ values (10, 15, and 20%) at the resonance condition and solar cells without AuNPs at the wavelengths of 500 and 610 nm (Figure 5). By altering the values of f_s_ to 10, 15, and 20% for AuNPs with a radius of 7.5 nm, we observed corresponding short-circuit current densities of 35, 50, and 67 A/m^2^, respectively (Figure 5A). These values were higher than those without AuNPs at wavelengths of 500 and 610 nm which are 34 and 4.5 /m^2^, respectively. For AuNPs with a radius of 10 nm and light intensity of 0.26 mW/mm^2^ for different values of f_s,_ 10, 15, and 20%, the short-circuit current densities were 35, 51, and 67 A/m^2^, respectively. Likewise, these values were higher than those of the bare active layer at the wavelengths of 500 and 610 nm, which were equal to 22 and 3.1 A/m^2^, respectively (Figure 5B).

**FIGURE 6:**
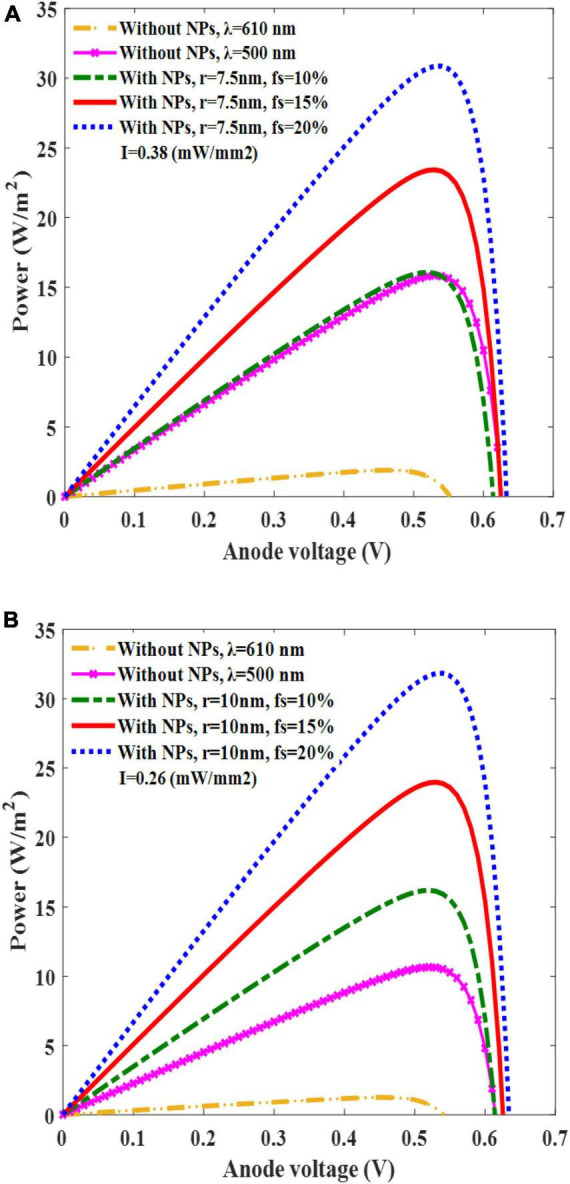
Plots of solar cell’s power versus anode voltage for different filling fraction (f_s_) of AuNPs in the active layer (10, 15, and 20%) and for a AuNP radius of 7.5 nm and a light intensity of 0.38 mW/mm^2^ **(A)**; corresponding results for a radius of 10 nm and a light intensity of 0.26 mW/mm^2^ **(B)**.

The power–voltage relationship was investigated to determine the solar cell performance parameters (Figure 6). The power–voltage graph of the solar cell versus the anode voltage for various f_s_ is presented for two different active layers: one with a radius of 7.5 nm and a light intensity of 0.38 mW/mm^2^ (Figure 6A), and the other with a radius of 10 nm and a light intensity of 0.26 mW/mm^2^ (Figure 6B). Since the irradiated light wavelength is in visible range (610 nm), and threshold light intensities of our AuNPs OSC are higher than natural sunlight illumination on the retinal neurons, these light powers are not compatible with the patients who retain some natural light sensitivity. These intensities can be used only for the patients whose natural vision are completely degenerated and do not retain any light sensitivity (Leccardi et al., 2020). These figures demonstrate the significant impact of the AuNPs on the solar cell output power compared with the bare structure at wavelengths of 500 and 610 nm. The power generated by the solar cell increased from 16 to 32 W/m^2^ as the f_s_ increased from 10 to 20%. This increase in power was attributed to the higher number of AuNPs, which led to higher light absorption and free carrier generation. The power curves reached their maximum values at the anode voltage of 0.53 V for each case. Using the related equations, the solar cell performance parameters were calculated, and are presented in Tables 3, 4. The filling factor was calculated as P_max_/I_sc_V_sc_ and the PCE was calculated as P_max_/P_in_, where P_in_ is A × I_in_, A is the cell area, I_in_ is the incident light intensity, and P_max_ is I_max_ × V_max_. The results in the tables show that the PCE of the solar cell increased as functions of the f_s_ and radius of the AuNPs owing to the increase in the overall absorption cross-section and current generation.

**TABLE 3:** Performance parameters for solar cells without and with AuNPs in the active layer with a radius of 7.5 nm and light intensity of 0.38 mW/mm^2^.

Solar cell’s structure	J_SC_ (A/m^2^)	V_OC_ (V)	FF (%)	PCE (%)
Active layer without AuNPs, λ=610nm	4.5	0.55	76.5	0.5
Active layer without AuNPs, λ=500nm	34	0.63	74.7	4.2
Active layer with AuNPs, *r* = 7.5 nm, f_s_ = 10%	35	0.61	75.4	4.2
Active layer with AuNPs, *r* = 7.5 nm, f_s_ = 15%	50	0.63	73	6
Active layer with AuNPs, *r* = 7.5 nm, f_s_ = 20%	67	0.64	72.2	8.1

**TABLE 4:** Performance parameters for solar cells without and with AuNPs in the active layer with a radius of 10 nm and light intensity of 0.26 mW/mm^2^.

Solar cell’s structure	J_SC_ (A/m^2^)	V_OC_ (V)	FF (%)	PCE (%)
Active layer without AuNPs, λ=610nm	3.1	0.54	77.6	0.5
Active layer without AuNPs, λ=500nm	22	0.62	77	4
Active layer with AuNPs, *r* = 10 nm, f_s_ = 10%	35	0.62	73.7	6.1
Active layer with AuNPs, *r* = 10 nm, f_s_ = 15%	51	0.63	74.7	9.2
Active layer with AuNPs, *r* = 10 nm, f_s_ = 20%	67	0.64	74.6	12.3

According to the performance parameters in both tables, the solar cells with active layers containing AuNPs had higher PCEs than those without AuNPs. Table 3 shows that the ratio of PCE for the solar cell with an active layer with AuNPs and a radius of 7.5 nm and f_s_ values of 15 and 20% to the PCE of the bare active layer were 1.4 and 1.92, respectively. The ratios in Table 4 for the active layer with AuNPs and radii of 10 nm and f_s_ of 15 and 20% to the bare layer were approximately 2.3 and 3, respectively. This demonstrates the importance of AuNPs in increasing the efficiency of solar cells. Between these cases, AuNPs with a radius of 10 nm and f_s_ values of 15 and 20% were the preferred options for designing the final intended solar cell structure because of their low-light intensity and high-PCE ratio.

### 3.3. AuNP-embedded solar cells can stimulate neurons efficiently

Incorporating AuNPs into solar cells enhances light absorption owing to LSPRs and free charge generation. Using AuNPs in the active layer of the solar cell, the current generation is enhanced; therefore, the neurons can be activated with a lower light intensity or reduced solar cell size. In this section, we investigate the optical threshold intensity required to stimulate neurons in solar cells with and without AuNPs-embedded in their active layers. In addition, we confirmed the efficacy of the plasmonic solar cells as a neural stimulation system.

To demonstrate whether our plasmonic solar cell can trigger neural activation, we first defined a stimulation threshold for the targeted neuron, which represented the minimum electrical stimulation parameter required for activation. The threshold current density and its corresponding voltage were determined to be 33.63 A/m^2^ and −0.065 V, respectively (Figures 5A, B). This point serves as a criterion, where the area above it is the suprathreshold region that can trigger neural activation, while the area below it is the subthreshold region that cannot activate the neuron. Using this threshold point, the threshold light intensity used to produce a threshold current for neural activation was determined for two types of solar cells (solar cells with 10 and 7.5 nm AuNPs with f_s_ values of 15 and 20%, respectively). Our findings indicate that a solar cell with 7.5 nm AuNPs requires a light intensity of 0.38 mW/mm^2^, whereas a solar cell embedded with 10 nm AuNPs requires only 0.26 mW/mm^2^, thus maintaining the wavelength of light fixed at 610 nm to maximize the photocurrent in both cases (Figure 5). Stimulating the neurons using the bare active layer requires a light intensity of 0.38 mW/mm^2^ at a wavelength of 500 nm, which is higher than the light intensity of the 10 nm AuNP-embedded active layer.

**FIGURE 7:**
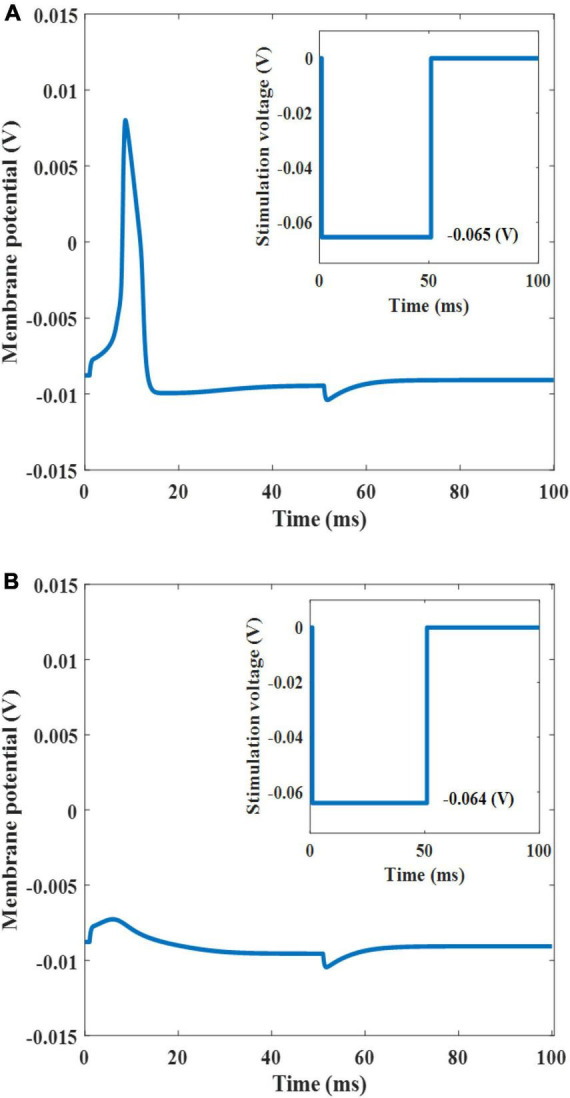
Stimulated neuron response (the inset shows the threshold input voltage of −0.065 V) **(A)**. Silent neuron response (the inset shows the under-threshold input voltage of −0.064 V) **(B)**.

When light is irradiated onto the plasmonic solar cell to produce a threshold cathodic voltage pulse (−0.065 V) with a duration of 50 ms, the neuron elicited an action potential (Figure 7A). However, when an input voltage of −0.064 V was applied (this value was slightly below the threshold level), the neuron remained silent (Figure 7B). As this threshold voltage lies within the water window, which is a potential range that does not induce water oxidation and reduction, stimulating neurons at this potential would not degrade the electrode (Boinagrov et al., 2016). Moreover, for our solar cell, the maximum voltage of the cathode was 0.64 V (which falls within the water window of 1.23 V) indicates that the voltage does not undergo electrolytic neural stimulation. To exclude this possibility, it is essential to determine accurately the actual voltage applied to the electrode and limit the voltage within the water window. First, using the impedance of the electrode–electrolyte interface (Z), the I–V relationship of the electrode was determined, as shown by the dotted purple in Figure 5. The intersection of the two I–V traces of the electrode and plasmonic solar cell represents the current and voltage that are actually applied to the neuron. For instance, when a light intensity of 0.26 mW/mm^2^ is applied to the 10 nm AuNPs with a f_s_ of 15%, it could deliver a voltage of 0.065 V and 33.63 A/m^2^ onto the neuron, which is within the water window.

In summary, our designed plasmonic solar cell efficiently stimulated neurons with a lower light intensity than solar cells without AuNPs. Moreover, we determined the maximum allowable optical intensity that would not induce water oxidation or reduction. We found that considering AuNPs with the radii of 7.5 or 10 nm using the light intensities of 0.38 and 0.26 mW/mm^2^, respectively, with f_s_ ≥ 15%, neurons were activated. Our main option is the case in which the radius of 10 nm owing to its lower intensity. The f_s_ values higher than 20% are not allowed because of the deteriorating active layer. The PCE ratios of these two types of AuNP-embedded active layers to the bare one was 2.3 and 3, respectively, thus revealing the high efficiency of this solar cell, which enables us to use either low-power illumination or a miniaturized cell structure. The simulation results indicate that this technique is suitable for highly efficient solar-cell-based retinal prostheses.

## 4. Discussion

In this study, we demonstrated that solar cells using bare active layers are not efficient enough to exploit either low-power incident light or high-current density to stimulate neurons. A high-current density enables the miniaturization of the cell dimensions to reach a high-resolution interface or to use low-power light illumination. In this way, by using the SIM4LIFE LIGHT simulator, we determined the current and voltage threshold levels required to stimulate the neurons. These threshold levels aid in designing and determining the desired characteristics of the solar cell interface. Subsequently, the active layer was modeled as a comb-shaped structure. We simulated a solar cell with and without spherical AuNPs, extracted the I–V curves of the cells, and compared two different conditions to determine the suitable characteristics of the solar cell. Our results indicate that a larger radius or f_s_ creates a higher current density. We adjusted these conditions to fulfill the threshold conditions of voltage and current density to stimulate the neurons. The power–voltage results are useful for calculating the performance parameters of the solar cells. The values of these parameters show that the preferred choices yield acceptable filling factor and PCE outcomes.

By comparing the absorption and scattering cross-sections, it was observed that for AuNPs with radii up to 10 nm, the absorption term is dominant, and the light absorption increases considerably. By increasing the radius of the AuNPs to values >10 nm, scattering is enhanced, but carrier trapping and exciton quenching increase and overcome scattering enhancement and reduce the PCE. The short-circuit current density data indicate that the resonant wavelength for AuNPs occurs at 610 nm and there is a discriminative enhancement for embedded active layers that have AuNPs with radii higher than 7.5 and 10 nm. The obtained results for a radius of 5 nm showed that either, (a) this size of AuNPs has weak resonance and could not stimulate neurons, or (b) required high-light intensities. In these simulations, we considered different AuNP radii (7.5 and 10 nm), which were illuminated at 0.38 and 0.26 mW/mm^2^, respectively. These are the optimum amounts used to fulfill the neuron activation conditions and are placed in the suprathreshold region of the I–V graph. The f_s_ value of 15% is the minimum filling fraction that satisfies the threshold point. f_s_ values >20% are not suitable and cause damage to the active layer. In contrast to the case with a radius of 7.5 nm and an intensity of 0.38 mW/mm^2^, the preferred case for embedded AuNPs corresponded to a radius of 10 nm and f_s_ values of 15 and 20% with an intensity of 0.26 mW/mm^2^, owing to its lower light intensity. In the preferred case, the ratios of PCE for the active layers with f_s_ values of 15 and 20% to the PCE of the bare active layer are 2.3 and 3, respectively, which allows us to miniaturize the device. These results show that these types of embedded AuNPs are suitable for solar cell design owing to their high PCE ratios and low intensities. Overall, this design is a modified structure for an efficient solar cell interface used as a retinal prosthesis.

## 5. Conclusion

The main purpose of this work was to improve the performance efficiency of solar cell-based retinal prostheses using embedded spherical AuNPs in the active layer and achieving higher power and current densities. The extracted outcomes showed that by illuminating the solar cells with incident light tuned to an LSPR wavelength of 610 nm, the photon absorption of the active layer was increased; this resulted in higher efficiency outcomes. This efficiency enhancement can be exploited to decrease the solar cell dimensions, which increases the retinal prosthesis resolution or decreases the light power. Our preferred values for the final structure are the AuNP radius of 10 nm, filling fractions of 15 and 20%, and a light intensity of 0.26 mW/mm^2^, which should be tuned to 610 nm. As discussed, with these values, the ratios of the PCE of the AuNP-embedded active layer in contrast to the bare one are 2.3 and 3, respectively, which can increase the solar cell efficiency and resolution by these proportions. The simulation results demonstrate that this structure can improve the resolution of the interface cells, which is a useful solution for enhanced solar cell-based retinal prosthesis performance.

## Data availability statement

The raw data supporting the conclusions of this article will be made available by the authors, without undue reservation.

## Author contributions

KE and AR: conceptualization and generating the idea, data collection, and drafting and revising. AR: analysis and writing. KE: funding. Both authors contributed to the article and approved the submitted version.
